# Spindle cell carcinoma of the lung with myogenic differentiation in a young female patient: a rare case report

**DOI:** 10.1097/MS9.0000000000003308

**Published:** 2025-04-22

**Authors:** Wahab Khan, Naima Kazi, Haider Ali, Burshra Noor, Shifa Haleem, Qaisar Ali Khan, Ravina Verma

**Affiliations:** a Khyber Teaching Hospital MTI KTH, Peshawar, Pakistan; b Khyber Girls Medical College, Peshawar, Pakistan; c Khyber Medical College, Peshawar, Pakistan; d Hayatabad Medical Complex, Peshawar, Pakistan; e St. George’s University School of Medicine, True Blue, Grenada

**Keywords:** lung tumors, sarcomatoid neoplasm, spindle cell carcinoma

## Abstract

**Background:**

Spindle cell carcinoma is a sporadic sarcomatoid neoplasm in the lung and pleura, comprising only 0.3–1.3% of all malignant lung neoplasms. This case highlights a rare case of aggressive spindle cell carcinoma in a young female patient with no risk factor.

**Case summary:**

A 20-year-old female patient with no other comorbidities presented with a chronic cough associated with hemoptysis and right-sided chest pain off and off. Auscultation of the lungs revealed decreased breath sounds over the left lung. X-ray chest and computed tomographic (CT) scan of the chest showed a round homogenous opacity in the left lung. A biopsy of the mass confirmed the diagnosis of spindle cell carcinoma with focal myogenic differentiation and infarctive necrosis.

**Case discussion:**

Spindle cell carcinoma is often difficult to diagnose histologically as it usually shows pleomorphism, exhibits minor differentiation, and includes components such as sarcoma, fusiform, or giant cells.

**Conclusion:**

Diagnosing spindle cell carcinoma of the lung could be challenging, especially in young patients with no other risk factors.

## Introduction

Sarcomatoid neoplasms in the lung and pleura are sporadic, accounting for about 0.3–1.3% of all malignant lung neoplasms^[1]^. They belong to a group with minor differentiation. Non-small cell carcinoma can have sarcoma, fusiform, or giant cell components. The World Health Organization classed it as pleomorphic carcinoma, spindle cell carcinoma, giant cell carcinoma, carcinosarcoma, and pulmonary blastoma^[1]^. Spindle cell carcinoma typically affects the oral cavity, larynx, breasts, kidney, uterus, conjunctiva, prostate, and other organs, with uncommon cases affecting the lungs. The majority of affected individuals are male (4:1) with a smoking history and an average age of 65 years^[2]^. The disease’s aggressive nature makes the stage the most reliable predictor of prognosis. Patients with this entity have a terrible prognosis, with a 5-year survival rate of around 20%^[3,4]^.

**HIGHLIGHTS**

- Spindle cell carcinoma is a type of sporadic sarcomatoid neoplasms in the lung and pleura, comprising only 0.3–1.3% of all malignant lung neoplasms.
- Diagnosing spindle cell carcinoma of the lung could be challenging, especially in young patients with no other risk factors.
- This case also highlighted the critical importance of early and accurate diagnosis to guide effective treatment.
- Given the tendency of tumors for rapid progression and high recurrence rate, timely surgical intervention remains vital, as conventional therapies like chemotherapy and radiotherapy often fall short in managing inoperable cases.

Histopathological diagnosis is crucial for characterizing and classifying these tumors. The tumor’s heterogeneity and pathological pleomorphism require complete removal for a conclusive diagnosis, while tiny samples can be used to suspect^[4,5]^. It can be difficult to diagnose, especially with small biopsies, because sarcomatoid mesothelioma, metastatic melanoma, and high-grade sarcomas may show overlapping morphological features. This is why immunohistochemistry is often used to evaluate the sample of thoracic tumors together with specific markers, becoming of great potential value^[6]^.

This case report aims to highlight the sporadic occurrence of spindle cell carcinoma, a rare and aggressive sarcomatoid tumor of the lungs. This case further contributes to the existing literature by demonstrating that sarcomatoid tumors can arise in young populations, even in the absence of risk factors. Through this case, we would have explored the response to surgery, chemotherapy, or radiotherapy, but the patient’s refusal of surgery and late presentation limited such information. This work adheres to the Surgical Case Report (SCARE) guidelines for reporting surgical case reports^[7]^.

## Case presentation

A 20-year-old female, a nonsmoker with no significant past medical history, presented to the pulmonology outpatient department at a tertiary care hospital in December 2024, with a chronic cough that had persisted for 4 months. Initially, the cough was dry but later became productive, with occasional streaks of frank blood. Over the last 2 weeks, the frequency of her cough increased, and it was associated with chest pain, which worsened with deep inspiration.

Upon further questioning, the patient reported a noticeable loss of appetite and unintentional weight loss of approximately 6 kilograms over the past 4 months. She denied any history of fever, night sweats, or recent exposure to individuals with tuberculosis. Additionally, she did not experience hoarseness of voice, and there was no history of recent travel or exposure to environmental pollutants, including smoke. The patient had no known allergies, and her family history was unremarkable for any pulmonary or oncological diseases. All the vaccinations were up to date. The patient used cough suppressant syrup and occasionally antibiotics and home remedies but the symptoms did not improve.

**Figure 1.**
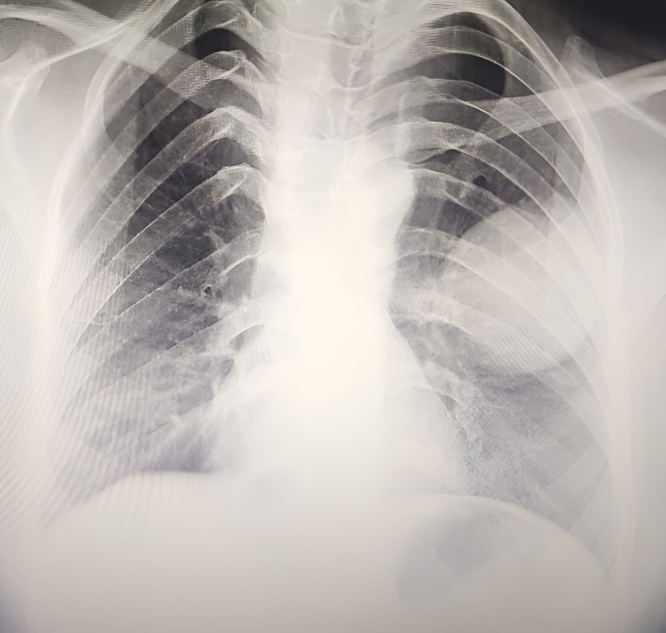
X-ray chest shows round homogenous opacity in the left middle zone of the lung.

The patient appeared cachectic with pale conjunctivae. Her vital signs were stable: blood pressure 110/70 mmHg, heart rate 87 beats per minute, respiratory rate 20 breaths per minute, temperature 98.6°F, and SpO2 95% on room air. On respiratory examination, chest wall movement was reduced on the left side, with decreased vocal fremitus, dull percussion in the left upper chest, and reduced breath sounds and vocal resonance on the left side. Crepitations were also noted at the left lung base. Initial laboratory investigations, including complete blood count (CBC), erythrocyte sedimentation rate (ESR), sputum acid-fast bacilli (AFB), and chest x-ray, were ordered the results are shown in Table 1 and Fig. 1.

**Table 1:** Initial laboratory investigations

Investigation	Result	Reference range
Hemoglobin	10 g/dL	12.0–14.0
Total leukocyte count	12 000 cells/µL	4–11 × 10^3^
Platelets	246 000 cells/µL	150–450 × 10^3^
C-reactive protein	34 mg/L	<5.0
Erythrocyte sedimentation rate	60 mm/h	<20
Sputum acid fast bacilli	Negative	

g/dL: gram per deciliter; µL: microliter; mg/L: milligram per liter.

**Figure 2.**
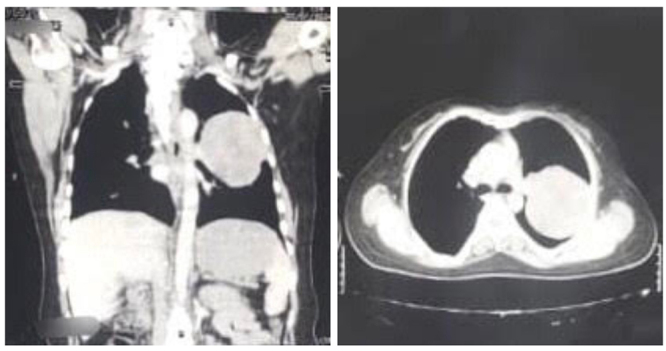
Computed tomographic scan of the chest showing heterogenous soft tissue attenuation mass in the left upper and lower lobe of the lung.

Based on the x-ray findings and negative sputum AFB, pulmonary malignancy was suspected. A computed tomographic scan of the lung was advised that revealed a heterogenous soft tissue attenuation mass in the left upper and lower lobe with patchy areas of perilesional ground glass haze as shown in Fig. 2.

**Figure 3.**
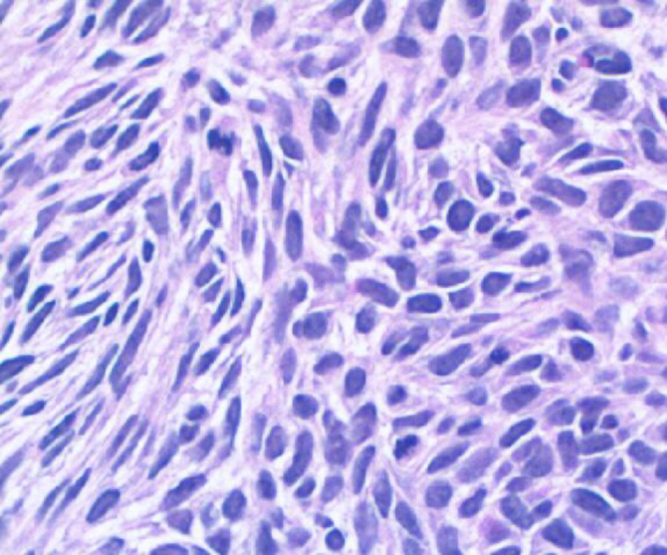
Histopathology of the specimen showing pleomorphic spindle cells.

A CT-guided transthoracic biopsy of the lesion was done which showed pleomorphic spindle cells with focal myogenic differentiation. Immunohistochemical staining was positive for Desmin and SMA as shown in Fig. 3.

The patient was referred to a surgical oncologist but she refused any surgical intervention. She was then referred to a medical oncologist for further management. She was started on doxorubicin, ifosfamide, and Mesna and was asked for a close follow-up with a positron emission tomographic (PET) scan. The PET scan revealed a large left lung mass (predominantly necrotic) with intimal enhancing and faintly FDG avid septations. No FDG avid mediastinal lymphadenopathy was noted. Two soft tissue density nodules were noted in the left retro psoas space at the level of the sacroiliac joint with slight misregistration of activity superiorly.

## Discussion

Relevant literature was searched using PubMed, Midline, and Scopus. Mesh terms were made by using keywords such as “lung cancer,” “sarcomatoid neoplasm,” and “spindle cell carcinoma.” Studies on spindle cell carcinoma’s epidemiology, diagnosis, and management were searched. Other case reports were searched to see how our case report is different from theirs. Spindle cell (or sarcomatoid) lesions encompass more than 16 types of non-neoplastic, benign, and malignant entities in the upper aerodigestive tract. Accurately distinguishing between these subtypes is challenging, frequently resulting in either overtreatment or misdiagnosis of malignancy. Spindle cell carcinoma consists of a pure population of spindle cells, frequently involving the lung’s periphery.^[5,6]^

Spindle cell carcinoma’s clinical appearance, diagnosis, and treatment are largely unknown. Overall survival in a patient with spindle cell carcinoma is determined by the patient’s age, disease site, and stage at diagnosis^[7]^. Risk factors for spindle cell carcinoma include a strong correlation with smoking, with reported rates as high as 96.6% similarly, radiation exposure has staked its association with spindle cell carcinoma^[8]^. In addition, the average age of onset is 60 years; it is more common in men than in women and is strongly and recurrence is likely to occur early after surgery^[5,6,8]^. Our case is unusual, as it involves a 20-year-old female patient without any history of smoking or exposure to radiation, which deviates from the typical demographic profile.

Pulmonary spindle cell carcinoma frequently exhibits nonspecific clinical symptoms and radiologic imaging that overlap with other lung cancers. Patients often present at advanced stages or with symptoms affecting their daily lives. Typical symptoms include shortness of breath, persistent cough, hemoptysis, chest pain, voice changes or hoarseness, weight loss, fatigue, and headaches^[9]^. These manifestations were observed in our patient as well.

Radiographic features associated with spindle cell carcinoma lesions have been observed, but the data available remains limited. Radiologically, spindle cell carcinoma of the lung typically presents as a solitary large lesion, ranging from 2 cm to 18 cm, frequently located peripherally in the middle lobe. Vascular invasion is observed in 40–70% of cases^[10]^. Preoperative diagnosis is challenging, with approximately 60% of cases remaining undiagnosed due to the diverse morphological features, making FNAB alone insufficient for accurate diagnosis. CT imaging commonly reveals a peripheral cavitary lesion, with many spindle cell malignancies originating in the upper lobe. Macroscopically, central necrosis and hemorrhage in spindle cell carcinoma of the lung are larger than 5 cm^[10]^. However, in our patient, the CT scan identified a heterogeneous soft tissue attenuation mass in both the left upper and lower lobes.

We further depended on histopathology analysis for diagnosis. Spindle cell carcinoma, a variant of squamous cell carcinoma, is characterized by epithelial pleomorphic or spindled cells. As demonstrated in Fig. 3, the tissue sample from our patient exhibited spindle cell proliferation with focal myogenic differentiation. Immunohistochemical analysis revealed consistent staining with smooth muscle markers, including desmin and SMA. Initially, surgical intervention was deemed necessary for this patient; however, she declined the procedure. Consequently, the treatment plan was adjusted to include chemotherapy and close monitoring, upon follow up her PET scan revealed a large left lung mass (predominantly necrotic) with intimal enhancing and faintly FDG avid septations.

Spindle cell carcinoma is an aggressive tumor with a treatment approach similar to that of other non-small cell lung cancers (NSCLC), depending on the stage. Treatment options may include surgical resection, chemotherapy, and radiotherapy. Recently, drugs like programmed death 1 (PD1) and programmed death ligand 1 (PD-L1) have shown effectiveness against PSCC^[11,12]^. A database study conducted by Li et al. showed that the median survival of PSCC is about 4 months, with a 20% overall survival rate within 5 years. Patients undergoing surgery had longer survival compared to those receiving radiotherapy and chemotherapy^[13]^. Reports indicate that chemotherapy and radiation therapy are often inadequate for inoperable cases, highlighting the importance of early diagnosis and prompt surgical intervention for the effective management of spindle cell carcinoma^[14,15]^. This study represents the perspective of a single patient; therefore, further studies should be conducted to explore genetic tendencies, responses to various treatment options, and the overall survival rate of patients with PSCC.

## Conclusion

Our case report emphasizes the diagnostic complexity and rarity of spindle cell carcinoma, particularly in a young female patient without typical risk factors. Early and accurate diagnosis is needed to guide the proper treatment for this aggressive tumor. Given the tendency of Spindle cell carcinoma for rapid progression and high recurrence rate, timely surgical Intervention remains essential, as conventional therapies such as chemotherapy and radiotherapy often inadequately address inoperable cases.

## Data Availability

The datasets supporting the conclusions of this article are included within the article.

## Research registration unique identifying number (UIN)

Researchregistry (researchregistry10917).
